# A novel approach to flow visualization through mechanical heart valves

**DOI:** 10.1177/09544119251342868

**Published:** 2025-06-25

**Authors:** Dylan Goode, Ruby Dhaliwal, Jaymes Schmidt, Kibret Mequanint, Hadi Mohammadi

**Affiliations:** 1 Faculty of Applied Science, School of Engineering, The Heart Valve Performance Laboratory, University of British Columbia, Kelowna, BC, Canada; 2 Department of Chemical and Biochemical Engineering, The Western University, London, ON, Canada

**Keywords:** Heart valve, prosthetic heart valves, aortic valves, cardiovascular engineering, medical devices, simulation, heart simulators, flow visualization

## Abstract

Mechanical heart valves (MHVs) are indispensable in managing valvular disease, yet they often lack the hemodynamic efficiency of native valves and require lifelong anticoagulation therapy to mitigate thrombus formation. This study introduces a novel bileaflet mechanical heart valve (BMHV), the iValve, designed to address these challenges by more closely emulating native valve performance. Central to this research is the development of a custom-built steady-state flow simulator, which provides a cost-effective and innovative approach to visualizing flow dynamics through MHVs. Unlike traditional methods, this simulator allows for detailed observation of flow patterns, focusing on critical regions such as the central flow and hinge areas.

Using the novel flow simulator, the flow through the iValve was compared to that of conventional BMHVs, including the SJM/Abbott Regent and On-X valves. The iValve exhibited significantly reduced flow disturbances and vortex formation in the central flow region and effective hinge washing during the forward flow phase. These preliminary findings suggest that the iValve design minimizes energy loss and shear stress on blood elements, potentially reducing or eliminating the need for anticoagulation therapy. The steady-state flow simulator proved invaluable in these assessments, offering precise, qualitative insights into flow behavior that would be challenging to achieve with other methods. Future work, including pulsatile flow simulations and in vivo testing, will further explore the iValve’s clinical potential and validate these promising results.

## Introduction

Prosthetic heart valves are categorized as (1) bioprosthetic valves made from animal tissue,^1^ (2) transcatheter valves with collapsible bioprosthetic tissue,^2^ and (3) mechanical valves made from synthetic materials. Bioprosthetic valves provide excellent hemodynamics^3^ but typically deteriorate structurally after 7–8 years.^4^ Transcatheter valves, while minimally invasive, face similar durability challenges. Mechanical valves are highly durable but lack the hemodynamic efficiency of native valves. Merging the advantages of bioprosthetic and mechanical valves could transform cardiovascular treatments.

Bileaflet mechanical heart valves (BMHVs) have been the standard for mechanical valves since the St. Jude Medical (SJM) Regent valve was introduced in 1976.^5^ Current models, such as the SJM/Abbott Regent and On-X valves,^6^ feature two leaflets that obstruct central flow, causing flow separation, reversal, and energy loss. BMHVs also require anticoagulation therapy to prevent thrombosis due to the non-physiological shear stress in the hinge area^7^ and spikes in regional backflow velocity.^8^ These anticoagulants increase the risk of severe bleeding and blood clots if levels are insufficient.^9^ There is a critical need for a mechanical valve that mimics the performance of a healthy native valve.

This preliminary work presents a novel BMHV aimed at mimicking native valve performance more closely. These designs will be evaluated using an innovative hydrodynamic test system.

## Methods

### IValve design

Traditional BMHVs typically feature straight leaflets that pivot near the valve’s center, creating three orifices when fully open. In contrast, the iValve’s rounded, eyelid-like leaflets form a single central orifice. This elliptic design, inspired by studies that modified the SJM/Abbott Regent BMHV,^10,11^ has shown improved hemodynamic performance. The iValve’s saddle-shaped housing differs from the conventional circular designs, accommodating the unique leaflet actuation.

**Figure 1.**
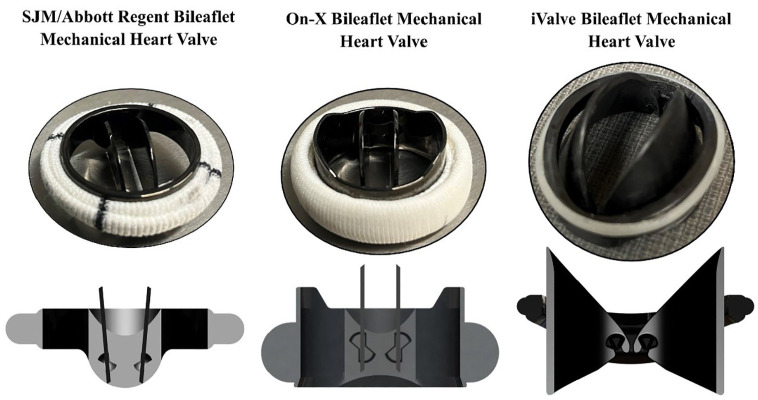
Sectional views of conventional bileaflet mechanical heart valves (the SJM/Abbot Regent Bileaflet Mechanical Heart Valve and the On-X Bileaflet Mechanical Heart Valve) and the novel iValve Bileaflet Mechanical Heart Valve. The difference in leaflet, housing, and hinge geometries is observed.

While most BMHVs use a butterfly hinge socket, the iValve allows leaflets to fully open to 90°, similar to the On-X BMHV (Figure 1).^12^ However, the iValve’s open hinge concept, with a pie-shaped appendage and a C-shaped socket, facilitates the washing of stagnant blood during lower shear forward flow, unlike conventional BMHVs that rely on high-shear flow during the closed phase. Though this process is necessary to remove the stagnant blood elements to prevent thrombus formation, it creates high shear rates that can be attributed to hemolysis and the initiation of the coagulation cascade.^13^ The iValve targets eliminating the need for hinge washing during the high shear closed phase by implementing a hinge geometry to remove blood elements during the lower shear forward flow phase. Comprehensive details about the iValve design are available in recent studies.^14,15^

### Steady-state flow simulation

A steady-state flow simulation was designed to evaluate flow patterns through the iValve and compare them to traditional BMHVs. While particle image velocimetry (PIV) systems are effective for visualizing flow, they are expensive. To overcome this, a novel kinematic flow simulator was developed to affordably visualize streamline patterns in BMHVs. The initial phase aims to establish a test system for steady-state flow and simulate peak systolic flow, with plans to later adapt for pulsatile flow. Flow patterns will be qualitatively analyzed under steady-state conditions.

**Figure 2.**
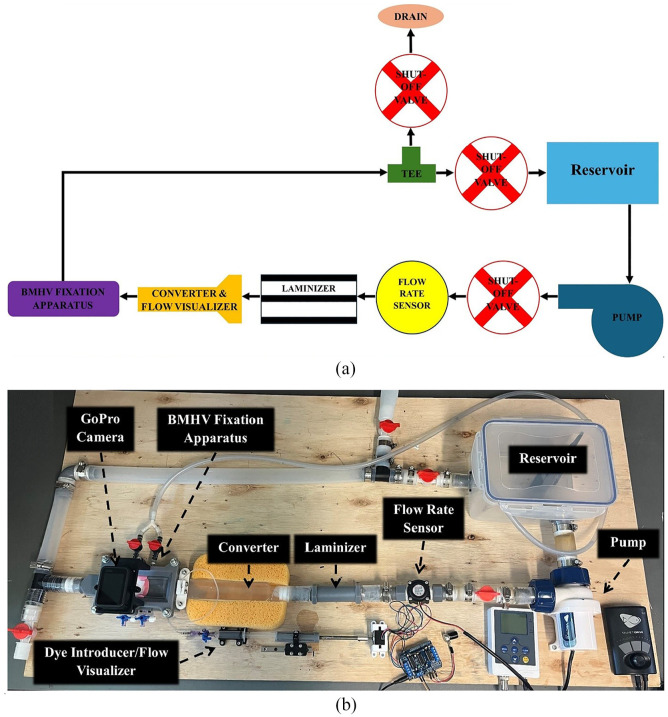
Steady-state flow simulation system: (a) Flow schematic, and (b) complete fabricated steady-state flow simulator experiment setup.

The steady-state flow simulator (Figure 2) includes several key components in a closed-loop circuit:

1. **Reservoir:** Stores the fluid volume for the system.
2. **Pump:** Circulates the working fluid within the system.
3. **Shut-Off Valves:** Control flow direction, regulate flow rates, adjust pressure, and isolate the reservoir for flushing.
4. **Flow Rate Sensor:** Ensures laminar flow by measuring flow rate.
5. **Laminizer:** Reduces disturbances from the pump and sensor to promote laminar flow.
6. **Converter and Flow Visualizer:** Converts the pipe configuration to a half-pipe and introduces dye to visualize streamlines and detect flow irregularities.
7. **BMHV Fixation Apparatus:** Anchors the BMHVs for evaluation.

To maintain fluid clarity the dyed flow exiting the fixation apparatus is redirected before reaching the reservoir. This minimizes mixing and ensures clear visualization of flow dynamics. When dye is not used, the flow is directed into the reservoir to conserve the working fluid during calibration and between experiments.

Flow visualization involves sectioning the BMHVs to fully expose the leaflets and hinges for detailed analysis. A camera records the flow through the BMHVs, concentrating on central and hinge flow areas. Central flow evaluates differences in flow patterns between conventional BMHVs and the iValve, while hinge flow focuses on comparing the novel iValve hinge to conventional butterfly hinges.

To minimize boundary effects, BMHVs are centrally positioned, with a thin-wall extrusion simulating the aorta to block backside flow. This setup ensures focused evaluation of hinge and central flow regions.

The footage is captured with a GoPro Hero 10 at 240 fps, 2.7K resolution, and a narrow lens.^16^ Distilled water is used to prevent bubbles, with a flow rate of 0.8 LPM ensuring laminar flow (Reynolds number 603). While pulsatile conditions typically have Reynolds numbers of 3000-4500,^17^ the steady-state simulator effectively visualizes laminar patterns. Blue Leakmaster Leak Locating Dye^18^ accurately traces flow with minimal mixing. Experiments are conducted at 22.5°C ± 0.5°C. Due to the qualitative nature of this preliminary work, this hydrodynamic study proved to be sufficient for the comparison of BMHVs, with future work utilizing a working fluid that more closely replicates blood. Further details on the hydrodynamic setup can be found in recent studies.^14^

## Results and discussion

**Figure 3.**
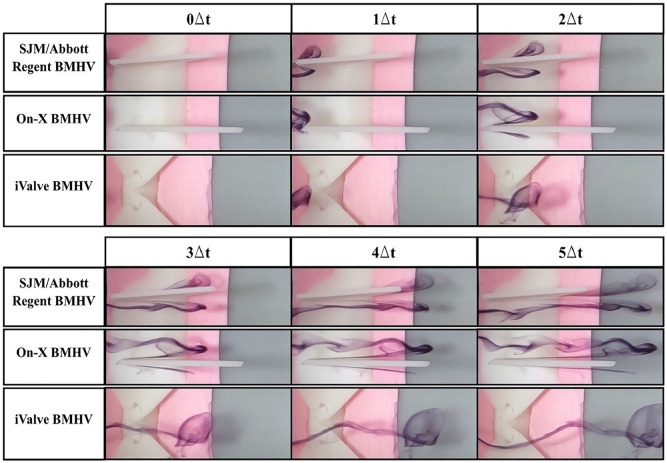
Central flow visualization comparison of sectioned bileaflet mechanical heart valves. The simulations were conducted at a flow rate of 0.8 LPM and Reynolds of 603. Distilled water is used as the working fluid at 22.5°C ± 0.5°C. Δt = 0.1 s.

**Figure 4.**
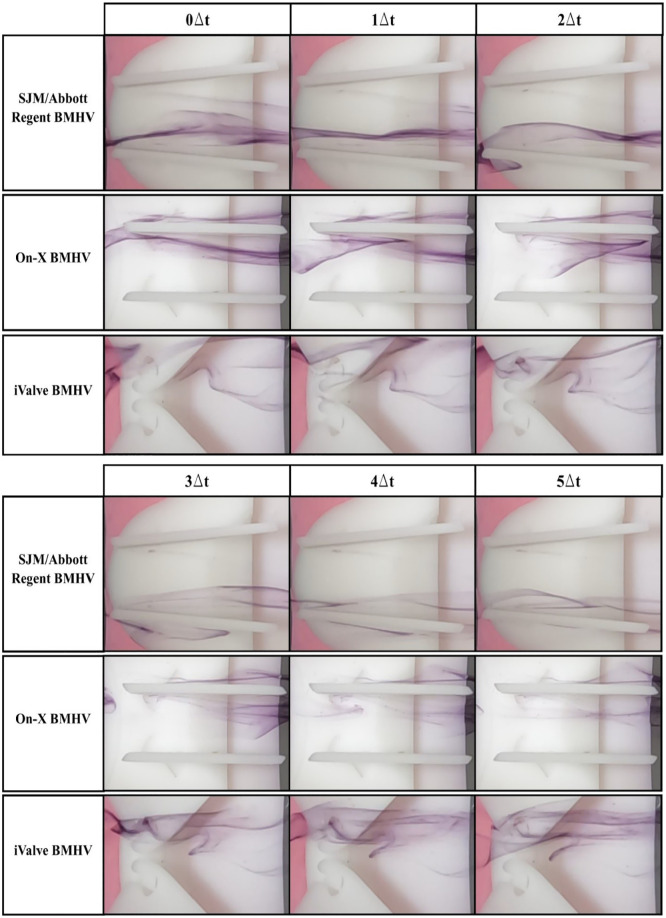
Hinge flow visualization comparison of sectioned bileaflet mechanical heart valves. The simulations were conducted at a flow rate of 0.8 LPM and Reynolds of 603. Distilled water is used as the working fluid at 22.5°C ± 0.5°C. Δt = 0.1 s.

**Figure 5.**
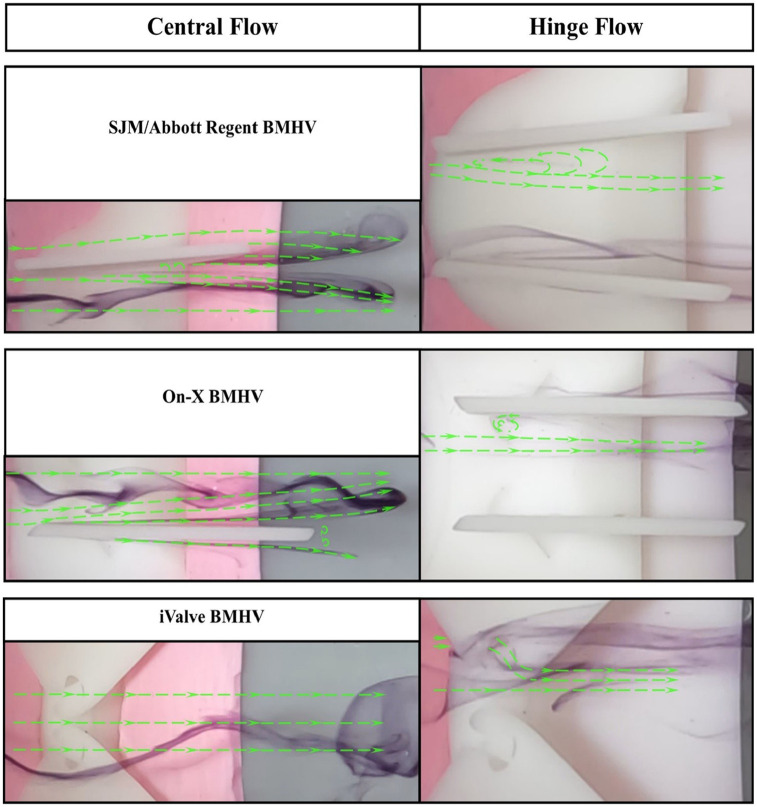
Perceived flow patterns of sectioned bileaflet mechanical heart valves. The simulations were conducted at a flow rate of 0.8 LPM and Reynolds of 603. Distilled water is used as the working fluid at 22.5°C ± 0.5°C. Δt = 0.1 s.

Using the steady-state flow simulator set to 0.8 LPM with distilled water at 22.5°C ± 0.5°C, the central and hinge flow areas of three BMHVs were analyzed. Central flow results are shown in Figure 3, hinge flow in Figure 4, and perceived flow patterns in Figure 5.

At 0.8 LPM and a Reynolds number of 603, dyed flow was introduced into the BMHVs. In the SJM/Abbott Regent and On-X BMHVs, the flow contacts the leaflets, forming wakes as it passes through. The SJM/Abbott Regent BMHV produces a significant wake due to its limited leaflet opening angle, causing energy loss and turbulence. Vortices form in the wake as the flow progresses, indicating turbulent flow and separation, consistent with prior PIV experiments.^19^

The On-X BMHV also forms a wake, though it is tighter, suggesting more streamlined flow. However, the presence of wakes still indicates energy loss, as these regions of recirculation and lower velocity contribute to a pressure drop across the valve.

The iValve BMHV exhibits a markedly different flow pattern. The dyed flow remains smooth and undisturbed as it passes through, retaining its initial geometry with minimal wakes or turbulence. This stable flow suggests the iValve design reduces energy loss and pressure drop, promoting efficient blood flow. The lack of wake formation highlights reduced flow disturbances, positioning the iValve BMHV as the most efficient in central flow dynamics compared to the On-X and SJM/Abbott Regent BMHVs.

Hinge flow analysis in Figure 4 requires detailed evaluation due to subtle flow visuals. The SJM/Abbott Regent BMHV shows minimal dyed flow within the hinge socket, but faint traces reveal reversed flow along the upper leaflet’s central orifice, forming an eddy. This eddy signifies energy loss, potentially causing high shear rates that can damage blood cells and elevate hemolysis and thrombus risks.

The On-X BMHV exhibits similar patterns, with stagnant dyed elements in the hinge area, highlighting the need for effective hinge washing during the closed phase to prevent high shear rates and clot formation. These patterns align with hinge socket flow dynamics observed in other BMHVs.^20^

The iValve BMHV demonstrates distinctly different dynamics. Flow entering the hinge area is smoothly redirected by the housing’s inflow edge, passing through the open hinge socket without stagnation. Frames 2Δt to 5Δt show dyed flow moving through the hinge without reversal, illustrating effective hinge washing during the forward flow phase. This open hinge design could reduce or eliminate the need for anticoagulation therapy by avoiding the high-shear washing required during the fully closed phase in conventional BMHVs.

## Conclusions

The steady-state flow simulator allows laminar flow visualization of streamlines through BMHVs in the forward flow phase. The approach to evaluating sectioned BMHVs provided valuable insight into how flow traverses the central and hinge areas. The iValve performed favorably against the conventional BMHVs, providing no flow disturbances or vortices in the central flow area. Also, there were no areas of flow stagnation in the hinge area, but rather observed was the hinge washing effect that would remove any stagnant blood elements trapped in the hinge socket. The combination of no flow disturbances in the central flow area and hinge washing during the forward flow phase would significantly reduce shear stress felt by blood elements. Reduced shear allows the feasibility to lessen the dosage of anticoagulation therapy and possibly be the first BMHV to eliminate the need for anticoagulation therapy. In vivo analysis will allow for the complete assessment of these theories.

The detailed flow visualizations from the steady-state flow simulator strongly support the iValve BMHV’s superior performance in central and hinge flow areas. This analysis underscores potential clinical benefits, including reduced shear rates and a decreased need for anticoagulation therapy. The steady-state flow simulator offers a novel, effective method for evaluating prosthetic valve flow patterns. Future enhancements, such as adapting the system to pulsatile flow, are recommended to better replicate physiological conditions.
